# Periprosthetic DXA after total hip arthroplasty with short vs. ultra-short custom-made femoral stems

**DOI:** 10.3109/17453670903074467

**Published:** 2009-06-01

**Authors:** Carlina V Albanese, Francesco S Santori, Laura Pavan, Ian D Learmonth, Roberto Passariello

**Affiliations:** ^1^Department of Radiological Science, Sapienza UniversityRome; ^2^Department of Orthopaedics, San Pietro Fatebenefratelli HospitalRome; ^3^Johnson and Johnson Medical SpA, DePuy DivisionPratica di Mare (RM)Italy; ^4^Avon Orthopaedic Centre, Southmead HospitalBristolUnited Kingdom

## Abstract

**Background and purpose** Dual-energy X-ray absorptiometry (DXA) analysis of the 7 periprosthetic Gruen zones is the most commonly used protocol to evaluate bone remodeling after the implantation of conventional femoral stems. We assessed the value of DXA after cementless primary total hip arthroplasty (THA) by comparing the effect of progressive shortening of the stem of two femoral implants on periprosthetic bone remodeling using a specifically developed protocol of analysis with 5 periprosthetic regions of interest (ROIs).

**Patients and methods** Bone mineral density (BMD) was evaluated in 37 patients in the plateau stage, 3 years after THA. Two femoral implants featuring conceptually new designs and surgical technique were tested: types 1 and 2, characterized by extremely short stem and virtual absence of distal stem, respectively.

**Results** We found that progressive shortening of the femoral stem produces more proximal loading, which effectively preserves metaphyseal bone stock and increases periprosthetic BMD in the medial ROIs over time. In the type 2 group, higher absolute BMD values were observed in medial ROIs 4 and 5. No differences were found in ROIs 1, 2, and 3.

**Interpretation** This study shows the flexibility of DXA in adapting the protocol of periprosthetic analysis to the specific requirements of new implant designs, and it shows its high sensitivity in evaluation of the biological response of bone to changes in implant shape.

## Introduction

Many factors may affect bone remodeling after total hip arthroplasty (THA). The stem geometry is believed to play an important role in the load transfer to the femur and, consequently, in femoral remodeling (Hua and Walker 1995, Aamodt et al. 2001). It has been reported that a proximal-loading device with extended metaphyseal geometry (lateral flare) preserves bone mass and increases periprosthetic bone stock (Leali and Fetto 2004), and that changes in the pattern of proximal loading stimulate the formation of new bone trabeculae, which stream up to the level of the lateral flare (Walker et al. 1999). Radiographic studies (Leali et al. 2002) and biomechanical tests (Walker et al. 1999, Kim et al. 2001, Westphal et al. 2006b) have confirmed that this kind of proximal geometry provides effective initial and long-term stability, suggesting that stems could be made shorter than designs that do not incorporate the lateral flare feature. Following this philosophy, an original custom-made ultra-short femoral stem with extensive proximal load transfer was developed (type 1). The shape of this new component has two prominent and innovative features. The first is the marked reduction of the diaphyseal stem. The second is the presence of a well-defined lateral flare intended to conform to the lateral femoral endosteal surface. Based on the initially good performance of this new prosthesis, the implant design was modified further, with an almost complete absence of the diaphyseal stem (type 2) (Santori et al. 2006a, b, c). In the last 25 years, there has been increasing interest in bone densitometry measurements because periprosthetic measurements may allow the detection of bone remodeling that cannot otherwise be observed due to the limited sensitivity of conventional radiographs (Mirsky and Einhorn 1998). Due to improvements in software and technology, dual-energy X-ray absorptiometry (DXA) provides accurate measurement of total and regional periprosthetic bone mineral density (BMD) after THA (Trevisan et al. 1993, Mirsky and Einhorn 1998, Spittlehouse et al. 1998, Venesmaa et al. 2001). This method has been shown to be useful in evaluation of the redistribution of mechanical forces around the hip joint following implantation of a prosthesis, and in assessment of how the proximal femur remodels around the implant (Brodner et al. 2004). The efficacy of DXA in the evaluation of bone remodeling patterns associated with different stem geometries has also been reported (Gibbons et al. 1997, Spittlehouse et al. 1998, Albanese et al. 2006).

We assessed the value of periprosthetic DXA in the bone remodeling plateau phase 3 years after THA using a 5-regions of interest protocol of analysis adapted to the new implant designs.

## Patients and methods

### Patient population

All 37 consecutive patients who had received either a type 1 or a type 2 custom-made implant 3 years previously were included in this observational study. Patients were allocated into 2 consecutive groups. In the first group, the earlier developed design (type 1) was used, while in the second group the latest developed design (type 2) was implanted. The inclusion criteria were the year of operation and the diagnosis of hip osteoarthritis. Patients who were post-menopausal, who were markedly overweight or underweight, or who had a history of previous surgeries on the same hip, femoral fractures, metabolic bone diseases, use of steroids or other drugs affecting bone metabolism, or intraoperative cracks were excluded from the study. All participants gave written informed consent to have 4 DXA examinations and to be included in the study, which was approved by the local Ethics Committee of Sapienza University of Rome (31 May 2006, no. 5.6).

### Study protocol

**Figure 1.**
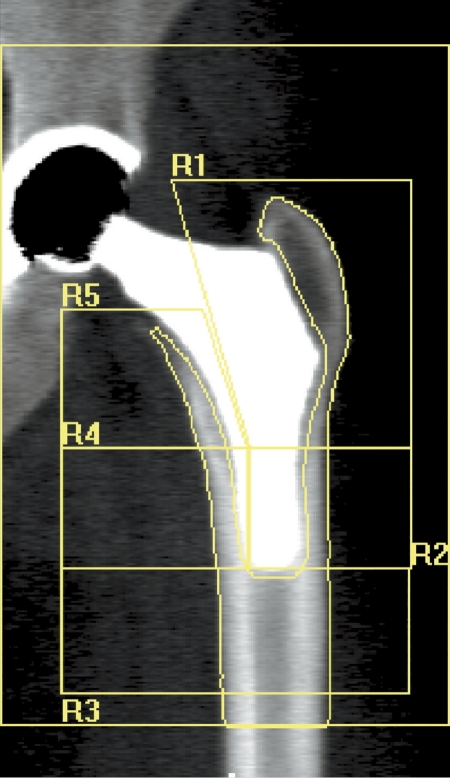
Type 1 custom-made femoral implant featuring an extremely short distal stem. DXA images of the proximal femoral periprosthetic analysis with 5 regions of interest (R1–R5).

**Figure 2.**
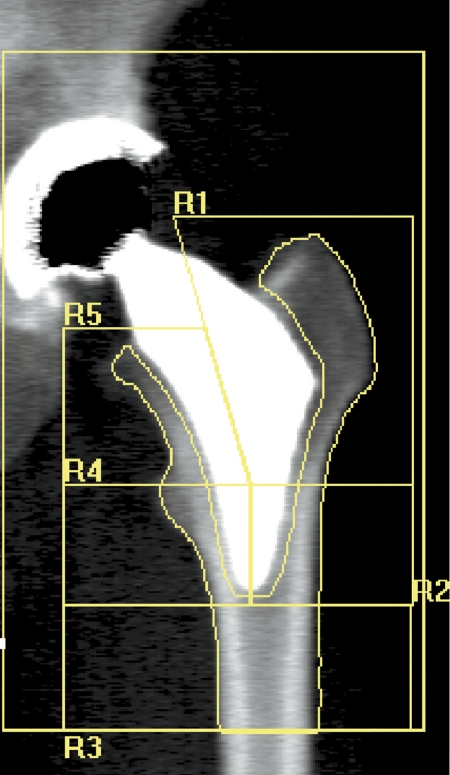
Type 2 custom-made femoral implant featuring an almost complete absence of the stem. DXA images of the proximal femoral periprosthetic analysis with 5 regions of interest (R1–R5).

Two slightly different new anatomical models of design were tested: the first model (type 1; Stanmore Orthopaedics, Stanmore, UK) was fully coated and featured an extremely short distal stem, which never extended more than 3 cm below the lesser trochanter (n = 16). The tip of the second model (type 2; DePuy International Ltd., Leeds, UK) never extended below the lesser trochanter (n = 21). Both models were cementless and had a large lateral flare (Figures 1 and 2). Preoperatively, each patient underwent a CT examination of the affected hip and a custom-made implant was specifically manufactured for each patient. The same surgeon (FSS) performed all operations in both study groups using the operative technique previously described by Santori et al. (2006c). At the time of the operation, the surgeon was provided with a single customized implant and a single corresponding broach. Standard radiographs were taken of all patients at the time of surgery and, postoperatively, at 1, 6, and 12 months, and annually thereafter as previously described (Santori et al. 2006a). The occurrence of stem subsidence and the appearance of radiolucent lines were recorded. A deviation greater than 2° from the longitudinal femoral axis was rated as either a varus or a valgus malpositioning. Clinical assessments were done using the Harris hip score. Periarticular calcifications were rated according to Brooker et al. (1973). All patients underwent partial weight bearing for 6 weeks and had a 3-month postoperative rehabilitation supervised by a physiotherapist. The height (cm) and weight (kg) of patients were measured.

### DXA technique and image analysis

DXA scans were performed using a Hologic QDR 4500-A device (Hologic Inc., Waltham, MA). The BMD (g/cm2) of the operated hip was measured using the “metal-removal hip” scanning mode. This mode has a higher resolution than the standard mode, giving a point resolution of 0.06 mm and a line spacing of 0.11 mm. The images were analyzed using the dedicated Windows analysis software (version 11.2). The patients were placed in supine position with the affected leg in slight internal rotation. The foot was secured in the Hologic foot positioning device in order to obtain reproducible rotation in all patients to limit measurement errors, since it has been demonstrated that rotation influences the BMD (Cohen and Rushton 1995, Mortimer et al. 1996, Martini et al. 2000). Due to the distinctive geometry of type 1 and type 2 implants featuring an almost complete absence of the stem, the conventional Gruen's zones were reduced from 7 to 5 (the conventional Gruen's zones 3 and 5 were eliminated). Thus, a 5-ROI protocol of analysis was developed (Figures 1 and 2).

The periprosthetic protocol of analysis was defined for the first patient to be examined with type 2 implant, then recorded and applied to all subsequent examinations in both groups in order to ensure an identical area for each ROI in both groups. The template of the protocol of analysis was neither increased nor decreased proportionally to the size of the implants and the size of the femur. In this protocol, the length of the implant was divided into 2 parts and subdivided into 2 lateral, 2 medial, and 1 inferior ROI. The 5 ROIs described in the protocol were defined as follows. ROI 1 (greater trochanter) includes the greater trochanter and starts from the ideal horizontal line drawn from the center of the lesser trochanter; ROI 2 (lateral) starts from the inferior edge of ROI 1 and ends at the tip of the implant; ROI 3 (below the tip) has the same height as ROI 2; ROI 4 (medial) has same bony landmarks and height as ROI 2; ROI 5 (calcar) includes the calcar and starts from the superior edge of ROI 4.

In order to compare the periprosthetic ROIs with the contralateral unoperated hip, the scan window was automatically reflected on the contralateral side and then adjusted. The BMD percentage of the contralateral, unoperated hip was calculated for each ROI as follows: (BMD of operated hip / BMD of unoperated hip) × 100.

The amount of BMD was recorded for each ROI independently in both hips. The overall BMD was summarized in the ROI's net average (NETAVG). To evaluate the influence of possible preferential weight bearing upon BMD after surgery, or individual differences in BMD, the contralateral proximal femur (total hip) and lumbar spine (L1-L4) were also measured in all participants.

DXA precision was assessed on 15 subjects with unilateral THA (6 women and 9 men; mean age 53 (31–74) years). All subjects underwent sequential DXA examinations of the periprosthetic hip, the contralateral unoperated hip, the lumbar spine, and the proximal femur—taken on the same day and measured twice, with repositioning between scans. Precision error was expressed as the coefficient of variation percentage (CV%). The CV% was calculated according to Aldinger et al. (2003). The precision (Table 1) was consistent with the literature (Aldinger et al. 2003, Shetty et al. 2006). The precision of lumbar spine and proximal femur was 1.2% and 1.3%. Additional quality controls were done every morning for the DXA equipment according to the manufacturer's guidelines, to verify the stability of the system, and did not show any shift or drift during the entire study period. The device used in our study was therefore characterized as stable. The same observer (CVA) analyzed all DXA examinations.

**Table 1.** Regions of interest (ROIs) precision error of the periprosthetic (operated) and contralateral (unoperated) hips.

ROIs	1	2	3	4	5	Mean (SD)	Net average
Periprosthetic	2.8	2.1	1.9	2.7	3.4	2.6 (0.8)	2.7
Contralateral	2.5	3.7	1.5	2.6	2.4	2.5 (0.4)	2.6

Precision error is expressed as the coefficient of variation percentage (CV%).

### Statistics

A Student's t-test was used to test the hypothesis of a difference between the group means. To test the assumption of homogeneity of variances and normality, Student's t-test was used at the 1% level (P < 0.01) alongside visual inspection of residuals from the mean. If age or other parameters showed a statistically significant difference between the two groups, an ANCOVA model was applied with age, or other parameters, as a covariate. Where the assumptions of the t-test were not met, the Wilcoxon rank sum test was applied. When the assumptions of the variables failed, the Mann-Whitney U test was used. The hypothesis tests were carried out with an alpha significance level of 5% (P < 0.05). The Bonferroni adjustment was used to maintain this level at 5%. Each p-value related to the primary endpoint was compared to the 1% significance level (0.05 divided by number of ROIs [5]) in order to maintain the overall alpha significance level. In all cases where the sample sizes were deemed too small, no analysis was carried out and descriptive summaries were examined. Data analysis was performed with SAS version 9.1.3 (SAS Institute Inc., Cary, NC).

## Results

Due to the small number of cases in each group, females and males were evaluated together. Type 2 patients were younger than those with type 1 implants (p < 0.001). Anthropometric parameters were equally distributed in both groups. No differences in L1-L4 and total hip BMD were observed between the study groups (Table 2).

**Table 2.** General characteristics of the study population. Values are expressed as number or mean (SD).

	Type 1	Type 2	p-value
Number	11	26	
Sex (M/F)	9/2	22/4	
Age, years	63 (10)	50 (8.7)	0.001
Weight, kg	73 (11)	78 (11)	0.2
Height, cm	173 (8.8)	173 (8.5)	0.9
BMI	25 (4.4)	26 (3.8)	0.4
Total hip BMD (g/cm^2^)	1.24 (0.10)	1.28 (0.14)	0.4
L1–L4 BMD (g/cm^2^)	1.32 (0.06)	1.21 (0.29)	0.5

BMI: body mass index; BMD: bone mineral density.

The Harris hip score improved from 43 and 47 to 95 and 96 points at the 3-year follow-up in the type 1 and type 2 groups, respectively. No thigh pain was reported at any of the postoperative evaluations.

There were no signs of radiographic subsidence or radiolucent lines in any of the stems. On standard radiographic assessment, stem alignment appeared to be neutral in all cases. No heterotopic ossification was observed.

Periprosthetic NETAVG revealed no statistically significant differences between the groups. In the type 2 group, higher absolute BMD values were observed in medial ROIs 4 (p < 0.001) and 5 (p = 0.004). No differences were found in ROIs 1, 2, and 3. ANCOVA adjusting for the difference in age between the two study groups provided the same conclusion as the main analysis (Table 3).

**Table 3.** BMD (g/cm^2^) of the 5 ROIs and BMD net average of the operated hips. Values are mean (SD).

	Type 1 (n = 11)	Type 2 (n = 26)	(95% CI)	p-value ^a^
ROI 1	0.74 (0.07)	0.82 (0.13)	(-0.01–0.19)	0.08
ROI 2	1.21 (0.23)	1.37 (0.26)	(-0.09–0.35)	0.2
ROI 3	1.61 (0.19)	1.58 (0.19)	(-0.20–0.14)	0.7
ROI 4	1.20 (0.19)	1.57 (0.19)	(0.18–0.51)	< 0.001
ROI 5	0.92 (0.13)	1.18 (0.21)	(0.09–0.43)	0.004
Net average	1.19 (0.09)	1.27 (0.14)	(-0.04–0.19)	0.2

^a^ ANCOVA model for between-group comparisons of BMD, with age as a covariate. The p-values have been compared to the significance level of 0.01 using the Bonferroni adjustement.

BMD: bone mineral density; ROI: region of interest;

With type 1, the implant caused bone loss in all ROIs compared to the contralateral hip, while in type 2 there was a gain in bone mass of 9.5% and 9.4% in regions 2 and 4, respectively. The differences in BMD percentage changes between the type 1 and type 2 groups were statistically significant in regions 4 (p = 0.001) and 5 (p = 0.007) (Table 4).

**Table 4.** Periprosthetic BMD percentage change between operated and unoperated contralateral hip of the 5 ROIs in the type 1 and type 2 groups. Values are mean (SD) or percentage (%).

	Type 1 BMD percentage	(n = 11) % change	Type 2 BMD percentage	(n = 26) % change	(95% CI)	p-value
ROI 1	87 (11)	–13	96 (13)	–4.0	(-3.8–21)	0.2 **^a^**
ROI 2	88 (14)	–12	110 (20)	9.5	(2.2–39)	0.3 **^a^**
ROI 3	100 (11)	–0.39	95 (6.2)	–5.2	(-14–1.2)	0.1 **^a^**
ROI 4	91 (13)	–9.0	110 (11)	9.4	(8.7–32)	0.001 **^a^**
ROI 5	76 (9.5)	–24	93 (17)	–6.7	(1.9–32)	0.007 **^b^**
Net average	99 (9.2)	–0.64	106 (8.2)	5.6	(-3.0–14)	0.2 **^a^**

^a^ ANCOVA model for between-group comparisons of %Contralateral, with age as a covariate.

^b^ Mann-Whitney U test for between-group comparison of %Contralateral.

The p-values have been compared to the significance level of 0.01 using the Bonferroni adjustement.

BMD: bone mineral density; ROI: region of interest; NETAVG: net average;

BMD percentage = (BMD of operated hip / BMD of non operated hip) x 100.

% change: percentage change between operated and non operated contralateral hip.

## Discussion

DXA is considered the most reliable tool to evaluate bone remodeling after THA using different stem designs (Albanese et al. 2006, Panisello et al. 2006). Analysis of the 7 periprosthetic Gruen zones is the most commonly used protocol to evaluate bone remodeling after the implantation of conventional femoral stems (Aldinger et al. 2003, Bodén et al. 2006, Panisello et al. 2006). In our study, all DXA measurements were taken 3 years after THA. Biomechanical adaptation to the prosthesis occurs mainly within 2–3 years, until a BMD plateau stage is reached (Mirsky and Einhorn 1998, Venesmaa et al. 2001, Aldinger et al. 2003, Brodner et al. 2004, Bodén et al. 2006, Panisello et al. 2006). The initial postoperative BMD (Venesmaa et al. 2001) or the preoperative BMD value can be used as the baseline value to estimate the change in periprosthetic BMD (Kobayashi et al. 2000). However, in cross-sectional studies the contralateral unoperated hip is also used to obtain individual comparative BMD values (Gibbons et al. 1997, Munting et al. 1997, Aldinger et al. 2003)—as we did.

The amount of periprosthetic bone remodeling appears to be influenced by different factors, including sex, age, weight, BMI, bone mass, and stem design. Many authors have reported conflicting results when testing factors that could possibly influence the periprosthetic BMD. Brodner et al. (2004) noted that gender affected Gruen zones 2, 3, 4, 5, and 6. They also observed that BMI influenced the BMD in zone 3 (lateral diaphysis) while age affected the greater trochanter (zone 1) and zone 4. Rahmy et al. (2004) reported that although preoperative BMD was the most important factor predicting bone loss after THA, stem design remains a major factor in influencing periprosthetic bone loss in Gruen zones 4 and 7, even after controlling for the effects of preoperative BMD and gender. Other authors (Korovessis et al. 1997, Sköldenberg et al. 2006) reported no correlation between age and periprosthetic changes in BMD while Kärrholm et al. (2002) evaluated multiple variables and found that postoperative BMD was only influenced by the stem design. In our study, type 2 patients were younger on average than type 1 patients. No differences were found between the 2 study groups in terms of radiological pre- and postoperative bone quality, anthropometrical parameters, and skeletal mineralization measured at the lumbar spine and contralateral proximal femur. Thus, the periprosthetic bone remodeling observed in these patients appears to be closely related to implant design rather than patient age.

We assessed the effect on bone remodeling of 2 metaphyseal implants with a design considerably different from that of most commercially available femoral stems. Type 1 had a very short stem, and type 2 had no diaphyseal stem. These prostheses were conceived and first implanted in 1995 by Santori et al. (2006a), the rationale of the new idea being based on the assumption that it is possible to achieve enough stability without the diaphyseal portion of the stem provided the implant has a well-defined lateral flare (Santori et al. 2006a, c).

Some publications on conventional femoral implants have reported a correlation between femoral stem size and proximal bone loss (Nishii et al. 1997, Sköldenberg et al. 2006), while other investigators have found no such correlation (Yamaguchi et al. 2000, Sychterz et al. 2001). Although a number of previous DXA studies have investigated periprosthetic bone remodeling following uncemented THA, a precise comparison of the results is not always possible due to differences in stem design and protocol. Our study represents the first experience on the effect on BMD of progressive shortening of the femoral stem in a short-stemmed prosthesis, evaluated by DXA. Increased bone mass in the stemless implant was found in medial ROIs 4 and 5 and in lateral and medial regions when compared to the contralateral unoperated femur. In the stemless implant group, calcar bone loss was only 7% as compared to 24% loss in the short-stem group. Previous studies on BMD changes in conventional stems have shown substantial bone loss in Gruen zone 7 (calcar). In the studies by Venesmaa et al. (2001) and by Rosenthall et al. (1999), bone loss in zone 7 at the 3-year follow-up was 23% and 20%, respectively. Rahmy et al. (2004) reported a mean loss of 16% in patients with an anatomic stem and 6% in patients with a more proximal loading implant. Munting et al. (1997) tested the behavior of a stemless femoral component and reported an increase in BMD in the proximal medial femoral cortex at the 3-year follow-up. In most femurs with an initial BMD that was lower than that of the unoperated hip, they also observed an increase in BMD until the BMD values became similar on both sides. In an experimental study, Decking et al. (2006) measured strain distribution before and after insertion of 3 different kinds of stems in human cadaveric femora: a conventional straight stem based on a distal anchorage concept, an anatomic stem designed to have proximal loading, and a “stemless” femoral neck prosthesis. They found a decrease in strain in the proximal femur after the insertion of both the straight and the anatomic stems, and a more physiological strain distribution in the medial region of the hip with the stemless implant.

An vitro study of the commercially available version that was developed of the type 2 prosthesis, which shares the design of this custom-made implant, suggested that poor bone quality, varus or valgus malpositioning, and implant undersizing, were important criteria that could lead to failure (Westphal et al. 2006). Thus, both surgical technique and careful patient selection appear to be crucial factors for the survivorship of an ultra-short implant. The positive clinical outcome observed in our study may be attributed to the experience of the operating surgeon. In fact, all baseline and follow-up radiographs showed correct alignment and sizing of the implant in both series. We cannot therefore evaluate the influence of potential varus or valgus misalignment on periprosthetic bone remodeling in such prostheses. However, Panisello et al. (2006) recently reported that in a conventional anatomic implant, malalignment had no clinical consequences and resulted in minimal differences in bone remodeling. Even so, despite the fact that implants were well aligned in both groups, the ultra-short implant showed better strain distribution—resulting in a more favorable pattern of bone remodeling in the ROIs known to be at high risk of bone loss. A similar finding was reported when this short implant was compared to different conventional prosthetic designs, characterized by a long or very long stem (Albanese et al. 2006).

Some limitations of this study should be considered. Because most subjects were men and the number of women was small, the effect of gender differences on bone remodeling was not evaluated. This work was an observational study that should be validated further in a prospective longitudinal trial. Single BMD evaluations have limited prognostic value, as the initial postoperative values are not known. Furthermore, use of the contralateral femur as a control does not necessarily account for inequality of BMD in the same patient because BMD at the start may not be identical in both hips. However, when data from time-consuming prospective studies are lacking, we have to use the unoperated side as a control. We believe nevertheless that this first report may enhance our understanding of bone remodeling behavior when a metaphyseal stemless prosthesis is implanted, and our work shows that DXA allows quantification of the bone mass around these new femoral implants.

In conclusion, our study suggests that DXA can be adapted to the specific requirements of a particular implant design. We suggest that a 5-ROI protocol of analysis is suitable for evaluation of bone remodeling after THA with stemless implants. Using this diagnostic technique and the 5-ROI protocol of analysis, we found that the ultra-short implant—which has a more anatomical proximal fit without having a diaphyseal stem with distal cortical contact—can provide immediate postoperative stability and a more physiological load distribution, thus increasing periprosthetic BMD in the medial regions over time.
